# Effectiveness of smoking reduction intervention for hardcore smokers

**DOI:** 10.1186/s12971-015-0034-y

**Published:** 2015-04-02

**Authors:** Tai Hing Lam, Yee Tak Derek Cheung, Doris Yin Ping Leung, Abu Saleh M Abdullah, Sophia Siu Chee Chan

**Affiliations:** School of Public Health, The University of Hong Kong, Hong Kong, China; The Nethersole School of Nursing, The Chinese University of Hong Kong, Hong Kong, China; Global Health Program, Duke Kunshan University, Kunshan, China; Duke Global Health Institute, Duke University, Durham, NC USA; Department of Medicine, Boston University Medical Center, Boston, USA; School of Nursing, The University of Hong Kong, Hong Kong, China

**Keywords:** Smoking cessation, Hardcore, Smoking reduction, Cigarette consumption, Quit attempt

## Abstract

**Background:**

The prevalence and correlates of hardcore smokers, who have high daily cigarette consumption, no quitting history and no intention to quit, have been studied in several western developed countries, but no previous trials of smoking cessation have tested intervention effectiveness for these smokers. The current study examined if hardcore smokers can benefit from smoking reduction intervention to achieve cessation, and explored the underlying reasons.

**Methods:**

*A posteriori* analysis was conducted on data from a randomized controlled trial of smoking reduction intervention on 1,154 smokers who did not want to quit. Odds ratios of 7-day point prevalence of abstinence, smoking reduction by at least 50% and quit attempt at the 6-month follow-up comparing subgroups of smokers were analyzed.

**Results:**

In hardcore smokers, the odds ratio comparing the quit rate between the intervention and control group was 4.18 (95% CI: 0.51-34.65), which was greater than non-hardcore smokers (OR = 1.58, 95% CI: 0.98-2.54). The number needed to treat for hardcore and non-hardcore smokers was 8.33 (95% CI: 5.56-16.67) and 16.67 (95% CI: 8.33-233.64), respectively. In smokers who did not have quit attempt experience and those who smoked more than 15 cigarettes daily, the odds ratio comparing intervention and control group was 3.29 (95% CI: 0.72-14.98) and 1.36 (95% CI: 0.78-2.36), respectively.

**Conclusions:**

The *a posteriori* analysis provided pilot results that smoking reduction intervention may be effective to help hardcore smokers to quit and reduce smoking. Having no previous quit attempt was identified as more important than having large cigarette consumption in explaining the greater effectiveness of the intervention.

**Electronic supplementary material:**

The online version of this article (doi:10.1186/s12971-015-0034-y) contains supplementary material, which is available to authorized users.

## Background

Smoking kills 6 million people each year in the world [1]. About 60% of adult current smokers in developed countries, however, do not want to quit or plan to quit in the near future [2]. Previous studies conceptualized hardcore smokers as those who have never thought to quit, have never taken any action to quit even if there is an easy way to do so, or have become totally discouraged from previous failed quit attempts [3]. There has been no universal definition for hardcore smokers, but three basic indicators of smoking characteristics were often used to specifically characterize hardcore smokers for epidemiological studies: (1) strong physical dependence of smoking, (2) no quitting history and (3) no intention to quit.

The strong tobacco control measures in Hong Kong have resulted in the lowest smoking prevalence in the developed world [4]. The prevalence of daily smoking in Hong Kong has been declining from 23.3% in 1982 to 10.7% in 2012 [5]. Using the representative data of smoking from the government, we found that the proportion of hardcore smokers increased from 21.8% in 2005 to 27.4% in 2008 [6], which was higher than several developed countries including the US [7], Canada [8], and England [9], and was comparable with Norway and Italy [10,11]. Therefore, Hong Kong is facing the challenge of increasing hardcore smokers coupled with declining smoking prevalence.

Several trials of smoking reduction intervention were conducted on smokers who did not want to quit, which showed that smoking reduction treatment does not restrain smokers to quit [12,13]. Conversely, the treatment with pharmacological assistance enhances the reduction and achieves eventual cessation outcomes [12,14-16]. A systematic review on smoking reduction treatment, including these trials, has concluded that the combination of pharmacologic and behavioral interventions was effective to increase quit rate for those who initially had no quitting intention [17]. Our Hong Kong randomized controlled trial of smoking reduction for smokers with no intention to quit found that smoking reduction intervention with nicotine replacement therapy (NRT) and behavioral counselling increased tobacco abstinence (Intervention group 17.0% versus Control 10.2%, p = 0.01) and reduction in cigarette consumption by at least 50% (50.9% versus 25.7%, p < 0.01, including quitters) at the 6-month follow-up [18]. Yet, its effectiveness for different sub-groups of smokers or hardcore smokers has not been examined. To the best of our knowledge, no studies have explored the smoking characteristics that might influence the effectiveness of smoking reduction intervention. The findings about the influential factors of quitting success in such trials can inform which kind of smokers can benefit from the quitting strategy through smoking reduction.

The current *a posteriori* analysis, based on our published RCT, aimed to examine if hardcore smokers can benefit from smoking reduction intervention to achieve cessation. The two research questions were: (1) Was the intervention of smoking reduction equally effective for hardcore and non-hardcore smokers to quit, attempt quitting or reduce smoking? (2) Did smokers without quitting experience or heavy cigarette consumption benefit from the smoking reduction intervention?

## Methods

### Data

The archived data of our published randomized controlled trial of a smoking reduction project on 1,154 smokers recruited during October 2004 to April 2007 were analyzed [18] (Clinical trial registration number: ISRCTN05172176 (http://www.controlled-trials.com)). All the participants were daily smokers who were not willing to quit but interested in reducing smoking. They were randomly allocated to two intervention groups and one control group (Additional file 1: Appendix A). Both intervention group A1 (n = 479) and A2 (n = 449) received 15-minute face-to-face counseling on smoking reduction by trained smoking cessation counselors and free nicotine replacement therapy (NRT) for eight weeks in total. The counseling emphasized the ultimate goal of complete cessation by focusing on the importance of smoking reduction, how reduction is useful and effective when quitting is difficult, and how to reduce (Additional file 2: Appendix B and Additional file 3: Appendix C). The former group (A1) also received a 3-minute counseling of adherence to NRT, which followed the guidelines on adherence interventions by the World Health Organization [19]. The control group B (n = 226) received a 10-minute brief advice on the health hazards of smoking and the importance of smoking cessation at baseline only. All the subjects were given a 12-page self-help quitting pamphlet, “Tips for Quit Smoking”, produced by Hong Kong Council on Smoking and Health.

### Outcome measures

Two primary outcomes at the 6-month follow-up, as in the original protocol, were used for assessing the efficacy of smoking reduction intervention on the hardcore and non-hardcore smokers: (1) self-reported 7-day point prevalence of tobacco abstinence, and (2) self-reported reduction by at least 50% in daily cigarette consumption compared with baseline. The third outcome, the rate of using NRT over 4 weeks at the 3-month follow-up, was not relevant here and thus excluded. In addition, self-reported quit attempt at the 6-month follow-up, defined as no smoking for at least 24 hours in the past 30 days, was included.

### Definition for hardcore smoker

As no universal definition of hardcore smoker is available, the following criteria which have been commonly used in previous studies were used to define hardcore smokers: (1) aged 26 years or above; (2) smoked daily for 5 or more years; (3) smoked 15 cigarettes or more a day; (4) had no intention to quit; and (5) had never attempted to quit [8,10,20-22]. The threshold for age and the years of smoking were included because youth smokers and smokers with short smoking history have not reached to a stable level of average daily assumption [3]. Although this definition of hardcore smokers has been criticized for its low predictive validity of future cessation [21] and the variability in the identification of hardcore smokers [8], they have been commonly used in epidemiological studies of hardcore smokers. All participants at baseline in this RCT met the 4^th^ criteria, hence the other 4 criteria were the determining criteria of hardcore smokers. Particularly noteworthy is that both hardcore and non-hardcore smokers in this study were all daily smokers who were not willing to quit but willing to participate in an RCT to reduce smoking. Also, we had very few subjects aged under 26 (n = 42, 4.3%) and had smoked for less than 5 years (n = 21, 2.2%). Therefore, the differentiating factors for hardcore smokers were “smoked 15 or more cigarettes a day” and “had made no quit attempt in lifetime”.

### Data analysis

Rates of abstinence, quit attempt and smoking reduction by at least 50% were compared between the intervention group (A1 and A2) and control group (B) with odds ratios and 95% confidence intervals adjusting for age group and sex. The results were computed by intention-to-treat approach such that all randomized subjects were included in the analysis, where those who were lost to follow-up were treated as failure to achieve the cessation outcome. The retention rate at 6-month follow-up for group A1, A2 and B was 89.1%, 94.4% and 95.6% (Additional file 1: Appendix A), so that the outcome discrepancy due to misclassifying those excluded subjects as failure would be small. Number needed to treat (NNT), which shows the number of treated subjects needed to have one additional successful outcome, was computed by taking the reciprocal of the risk difference between the intervention and control group [23]. The breakdown of the rates by hardcore or non-hardcore smokers, smoked or did not smoke 15 or more cigarettes a day, and had or had no previous quit attempt at baseline were analyzed to examine the effectiveness of the smoking reduction intervention for these sub-groups. To examine any interaction effect between intervention effect and types of smokers (hardcore versus non-hardcore, smoked versus did not smoke 15 or more cigarettes a day, and had versus had no previous quit attempt at baseline) on the cessation outcomes, a multivariate logistic regression was conducted to yield an odds ratio and p-value for the interaction term. Although p < 0.05 was considered as statistically significant, the objective of the *a posteriori* analysis was to provide ‘pilot’ or ‘proof of principle’ results for a larger RCT in the future.

### Ethics approval

The study was approved by the Institutional Review Board of the University of Hong Kong and Hospital Authority Hong Kong West Cluster (Ref no: UW 03–103 T/103).

## Results

**Figure 1:**
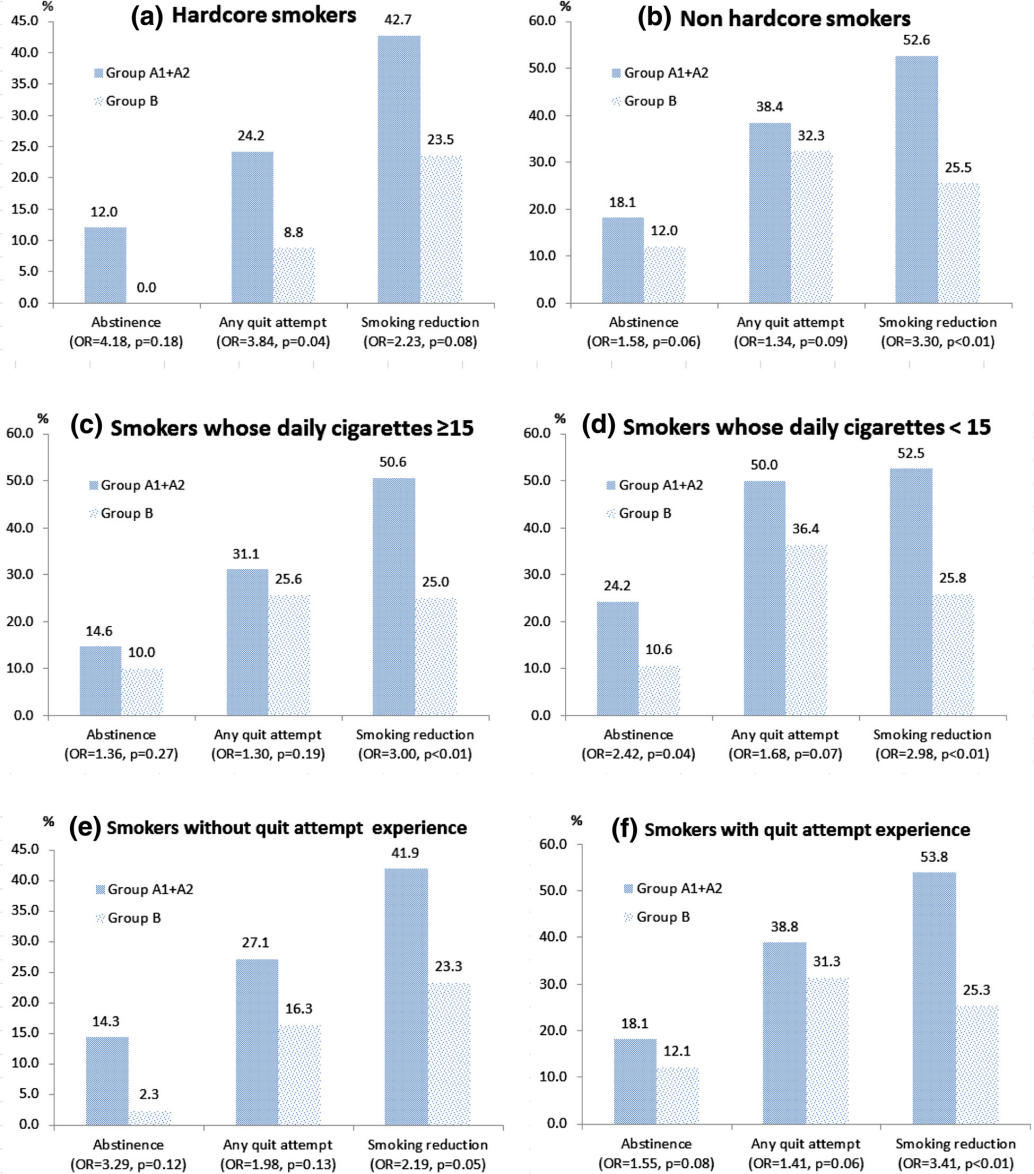
**Smoking cessation outcomes comparing intervention group (Group A1+A2) and control group (Group B) in (a) hardcore smokers; (b) non hardcore smokers; (c) smokers whose daily cigarette consumption equal to or greater than 15; (d) smokers whose daily cigarette consumption less than 15; (e) smokers without quit attempt experience; and (f) smokers with quit attempt experience at baseline.**

Additional file 4: Appendix D shows that hardcore and non-hardcore smokers were similar in all the socio-demographic characteristics. Figure 1 shows that the 6-month quit rate for hardcore smokers in the intervention group were 12%, but the control group was 0%. The odds ratio comparing the quit rate between the intervention and control group was 4.18 (95% CI: 0.51-34.65) in hardcore smokers, which was much greater than non-hardcore smokers (18.1% versus 12.0%, OR = 1.58, 95% CI: 0.98-1.88). The NNT for hardcore smokers was 1/0.12 = 8.33 (95% CI: 5.56-16.67), which was about half that for non-hardcore smokers (1/0.06 = 16.67, 95% CI: 8.33-233.64).

Hardcore smokers in this study had two major component criteria: (i) smoked 15 or more cigarettes a day and (ii) had no quit attempt before. Among those who smoked more than 15 cigarettes a day, the odds ratio of abstinence comparing the intervention and control group was 1.36 (95% CI: 0.78-2.36), which was smaller than that in those who smoked less (OR = 2.42, 95% CI: 1.03-5.65). (Figure 1) Among those smokers who had no reported previous quit attempt, the odds ratio was 3.29 (95% CI: 0.72-14.98), which was twice that in those who had quit attempt before (OR = 1.55, 95% CI: 0.95-2.53).

In hardcore smokers, receiving the intervention was associated with higher rate of quit attempt (24.8% versus 8.8%, OR = 3.84 (95% CI 1.07-13.85), p = 0.04). However, such difference was not significant in non-hardcore smokes (38.4% versus 32.3%, OR = 1.34 (95% CI 0.95-1.88), p = 0.09) and other sub-groups of smokers. Using smoking reduction by 50% as the outcome, the difference between the intervention and control group was similar in all the sub-groups of smokers. The odds ratios ranged from 2.23 to 3.41, which mean that the intervention group achieved better outcome of reducing smoking than the control group. As expected, probably because of insufficient statistical power, most of the interactions between group allocation and smoking characteristics in the models were not significant. (Table 1) There was no sufficient evidence that the effectiveness of the smoking reduction intervention was moderated by “hardcore” status, daily cigarette consumption and previous experience of quit attempt at baseline.

**Table 1:** **Analysis of the interaction effect on smoking cessation outcomes at the 6-month follow-up**

		**Odds ratio (95% CI)**	p-value*
**Interaction term**	**Group allocation X Hardcore status**		
Dependent variable	7-day abstinence	2.81 (0.34, 23.07)	0.34
Dependent variable	Any quit attempt (including quitters)	2.42 (0.67, 8.79)	0.18
Dependent variable	Smoking reduction by at least 50% (including quitters)	0.72 (0.29, 1.84)	0.50
**Interaction term**	**Group allocation X Smokers whose daily cigarette consumption ≥15**		
Dependent variable	7-day abstinence	0.56 (0.21, 1.53)	0.26
Dependent variable	Any quit attempt (including quitters)	0.73 (0.37, 1.46)	0.38
Dependent variable	Smoking reduction by at least 50% (including quitters)	0.96 (0.46, 1.97)	0.90
**Interaction term**	**Group allocation X Smokers with quit attempt experience**		
Dependent variable	7-day abstinence	2.15 (0.45, 10.17)	0.33
Dependent variable	Any quit attempt (including quitters)	1.34 (0.52, 3.42)	0.54
Dependent variable	Smoking reduction by at least 50% (including quitters)	0.68 (0.29, 1.59)	0.38

*p-value for interaction term: group allocation (intervention versus control) X type of smokers (hardcore versus non-hardcore).

## Discussion

This *a posteriori* analysis on data from an RCT has yielded new evidence on the effectiveness of smoking reduction intervention for hardcore smokers in increasing abstinence, quit attempt and smoking reduction. There was no significant interaction effect between group allocation and baseline smoking quantity, and between group allocation and quit attempt history. Hardcore smokers who received the smoking reduction intervention tended to have more abstinence and quit attempt than non-hardcore smokers who received the same intervention. Considering the outcome of quit attempt and reducing smoking by at least a half, the effectiveness of the intervention was not significantly different between smokers who had higher and lower cigarette consumption, and between smokers with and without quit attempt history.

The present study provided preliminary evidence that smoking reduction intervention may be effective in hardcore smokers, which is different from some population-level studies that light smoking quantity and high motivation to quit were predictive of abstinence [21,24,25]. The interaction between “hardcore smoker” status and “intervention” was not statistically significant probably due to the insufficient sample size, as the original RCT was not designed for the present sub-group analysis. But the odds ratios showed large differences between hardcore and non-hardcore smokers. It was mainly due to the very low quit and quit attempt rate in the hardcore smokers in the control group. The finding that hardcore smokers without receiving the intervention had zero quit rate after participating in an RCT can confirm the value of the notion of ‘hardcore’. Therefore, hardcore smokers can benefit from the smoking reduction intervention, with a smaller NNT being half of that for non-hardcore smokers. However, the statistical power for confirming the effectiveness of the intervention for the hardcore smokers was low due to the small group size and wide confidence interval. Future studies for exploring the effectiveness of various smoking cessation program among hardcore smokers with a large sample size are warranted.

Although the intervention emphasized reduction, about one in four hardcore smokers, who had no quit attempt experience and intention to quit at baseline, attempted to quit within the study period. The odds ratio of abstinence among smokers without a quit attempt was greater than those with the experience. On the contrary, the odds ratio of abstinence among smokers with higher cigarette consumption was smaller than those with lower cigarette consumption. It suggested that no quitting experience was more important than large cigarette consumption in explaining the greater effectiveness of the intervention. However, most previous population-level studies have supported that smokers without previous experience of quit attempt have a lower likelihood to have subsequent quit attempt than those who have such experience [24-26]. In this RCT, the smokers without quit attempt experience might be more empowered and encouraged to quit with the alternative approach of smoking reduction, as they did not want to achieve cessation through abrupt cessation.

Several studies have found that smoking reduction intervention with NRT and counseling are effective to help smokers without quitting intention [12,15,16,18,27-29]. Our results showed that around one-third of the smokers with daily cigarette consumption over 15 and one-fourth of the smokers who had no quitting experience attempted to quit smoking after receiving the intervention. The effectiveness was different, but not significantly, between smokers with higher and lower cigarette consumption, and with and without quit attempt experience, which was probably due to insufficient statistical power. These findings have added new knowledge that smoking reduction intervention helps smokers with hardcore smoking characteristics to quit using gradual cessation with free NRT. The hardening hypothesis expects that those who continue to smoke have an increasing resistance to social pressure to quit smoking [30]. Our findings have provided the first evidence (proof of concept evidence) that future RCTs with larger sample size to test whether such progressive approach of smoking cessation can help hardcore smokers to quit and reduce the hardening of the smokers are warranted. Although previous studies supported that reducing smoking do not undermine cessation motivation and effort [14,18], the existing clinical practice guidelines of treating tobacco use in the US do not recommend clinicians to suggest smoking reduction for smokers as such advice may decrease the proportion of smokers willing to make a quit attempt [31]. Our results suggested that smoking reduction intervention might be an alternative for hardcore smokers by motivating them to reduce smoking first and then quit.

The main strengths of the present study are that the data were from an RCT and the outcomes were assessed with blindness to the hardcore or non-hardcore status of the subjects. The RCT targeted smokers who had no intention to quit, in which the data had a large number of hardcore smokers to facilitate the present exploratory analysis. The main limitations were the *a posteriori* nature and sample size in categories, which limited the statistical power for implication. Also, all our subjects were willing to reduce and participate in the RCT. They might be “less hardcore” than smokers who never seek professional help for quitting. The findings of the greater effectiveness of smoking reduction on hardcore smokers are not definitive, but they can provide some proof of principle evidence to support further and larger RCTs on smoking reduction on hardcore smokers with the ultimate aim of quitting. Lastly, the current analysis relied on self-reported smoking status of the participants. Cotinine level in urine was measured in the self-reported quitters at the 6-month follow-up, but only 102 out of 181 quitters underwent the test, and, of them, 84 passed the test. The result from the biochemical confirmation was not used because of the low retention rate as majority of quitters refused the test.

## Conclusion

The *a posteriori* analysis of the RCT on smoking reduction intervention suggested that the intervention may be effective to increase the rate of abstinence, quit attempt and smoking reduction by at least 50% in hardcore smokers. Having no experience of quit attempt was more important than having large cigarette consumption to explain the greater effectiveness of the intervention. Smoking reduction intervention is an alternative to motivate hardcore smokers to reduce smoking first and then quit. Further research is needed to derive conclusions on the effect of smoking reduction for the hardcore smokers.

## Additional files

Additional file 1:
**Appendix A: CONSORT chart of the study.**
Additional file 2:
**Appendix B: Manual for behavioral counseling of the intervention Group A1 (Smoking Reduction + Adherence Counselling).**
Additional file 3:
**Appendix C: Manual for behavioral counseling of the intervention Group A2 (Smoking Reduction).**
Additional file 4:
**Appendix D: Baseline characteristics of the participants by hardcore smoking.**
