# Supplementary material for: Effectiveness of smoking reduction intervention for hardcore smokers
Source: Tob Induc Dis. 2015 Apr 2;13(1):9. doi: 10.1186/s12971-015-0034-y (PMC4391680; doi:10.1186/s12971-015-0034-y)
Supplement: Additional file 1: — Appendix A: CONSORT chart of the study. [file 12971_2015_34_MOESM1_ESM.docx]

Appendix A: CONSORT chart of the study

Allocated to Intervention A1 (n=479)

- Received allocated intervention (n=429, 89.6%)
- Did not receive allocated intervention (n=59, 12.3%)
- Lost to follow-up (n=52)
- Refused (n=7)

Randomized (n=1154)

Excluded (n=13809)

- Not meeting inclusion criteria (n=8578)
- Refused to participate (n=5231)

Assessed for eligibility (n=14963)

Analyzed (n=479)

Allocated to Intervention A2 (n=449)

- Received allocated intervention (n=389, 86.6%)
- Did not receive allocated intervention (n=60, 13.4%)
- Lost to follow-up (n=54)
- Refused (n=6)

Allocated to Control B (n=226)

- Received allocated intervention (n=226, 100%)

Further intervention at 1 month

- Received allocated intervention (n=381, 79.5%)
- Did not receive allocated intervention (n=98, 20.5%)
- Lost to follow-up (n=83)
- Refused (n=15)

Further intervention at 1 month

- Received allocated intervention (n=353, 78.6%)
- Did not receive allocated intervention (n=96, 21.4%)
- Lost to follow-up (n=80)
- Refused (n=16)

Follow-up at 3 month

- Attended follow-up (n=413, 86.2%)
- Lost contact (n=61, 12.7%)
- Refused (n=5, 1.0%)

Follow-up at 3 month

- Attended follow-up (n=385, 85.7%)
- Lost contact (n=55, 1235%)
- Refused (n=9, 2.0%)

Follow-up at 6 month Questionnaire

- Complete questionnaire(n=427, 89.1%)
- Lost contact (n=50, 10.4%)
- Refused (n=2, 0.4%)

Biochemical validation test

- Attended cotinine & CO test (n=59, 59.6%)
- Attended CO test (n=76, 29.8%)

Analyzed (n=449)

Analyzed (n=226)

Follow-up at 6 month Questionnaire

- Complete questionnaire(n=405, 94.4%)
- Lost contact (n=39, 9.1%)
- Refused (n=5, 1.2%)

Biochemical validation test

- Attended cotinine & CO test (n=31, 51.7%)
- Attended CO test (n=74, 33.8%)

Follow-up at 6 month Questionnaire

- Complete questionnaire(n=216, 95.6%)
- Lost contact (n=9, 4.0%)
- Refused (n=1, 0.4%)

Biochemical validation test

- Attended cotinine & CO test (n=12, 52.2%)
- Attended CO test (n=21, 36.8%)
