# Supplementary material for: Effectiveness of smoking reduction intervention for hardcore smokers
Source: Tob Induc Dis. 2015 Apr 2;13(1):9. doi: 10.1186/s12971-015-0034-y (PMC4391680; doi:10.1186/s12971-015-0034-y)

Appendix B: Manual for behavioral counseling of the intervention Group A1(Smoking Reduction + Adherence Counselling)
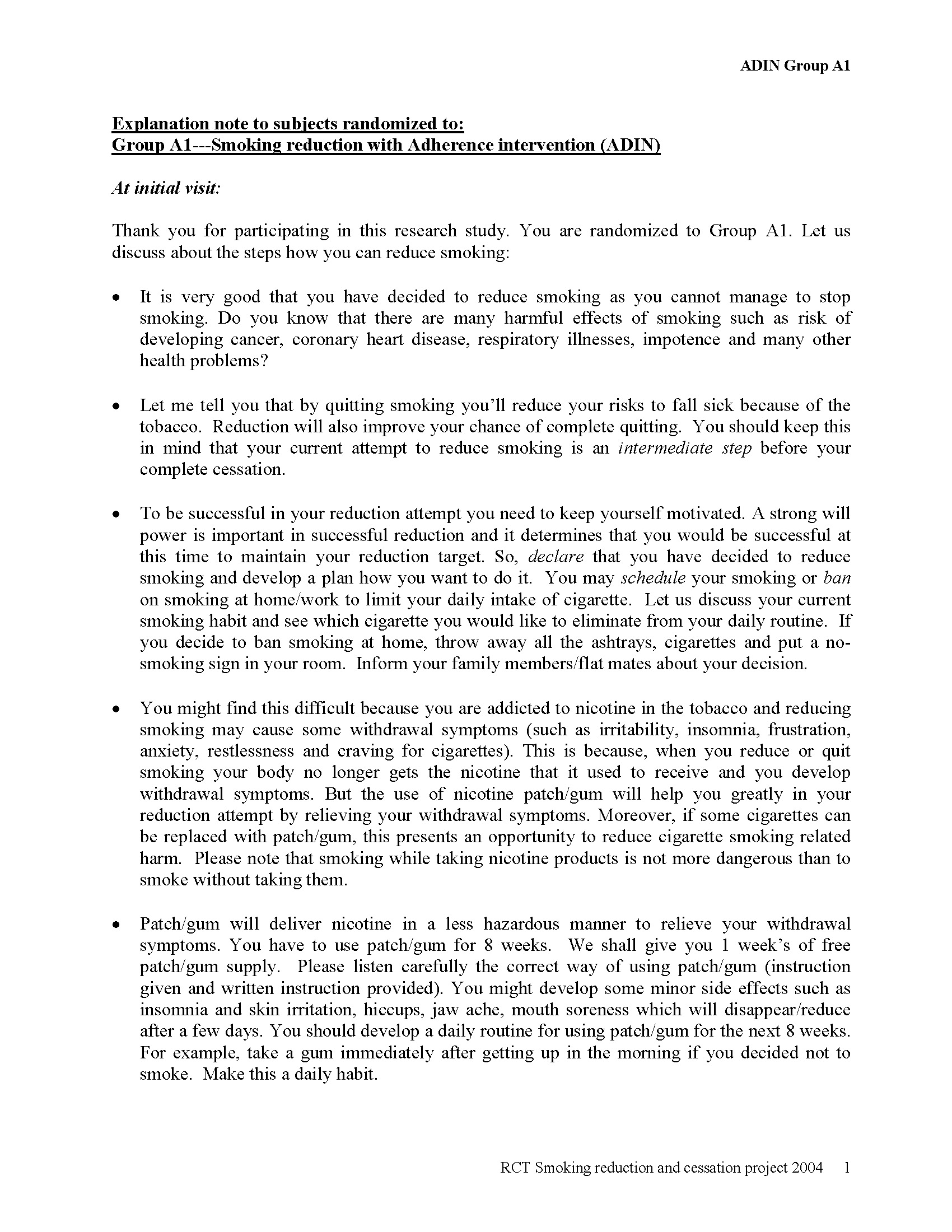


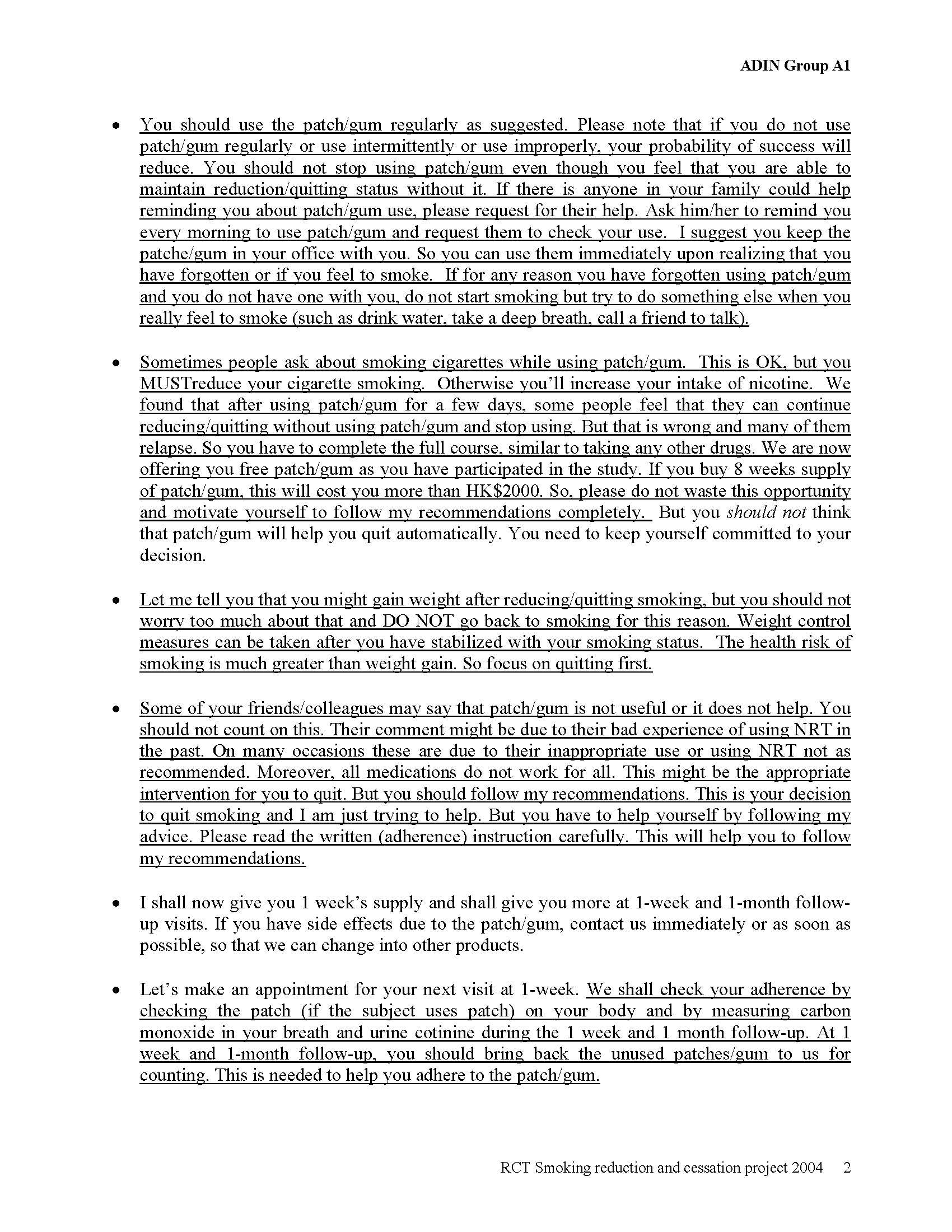


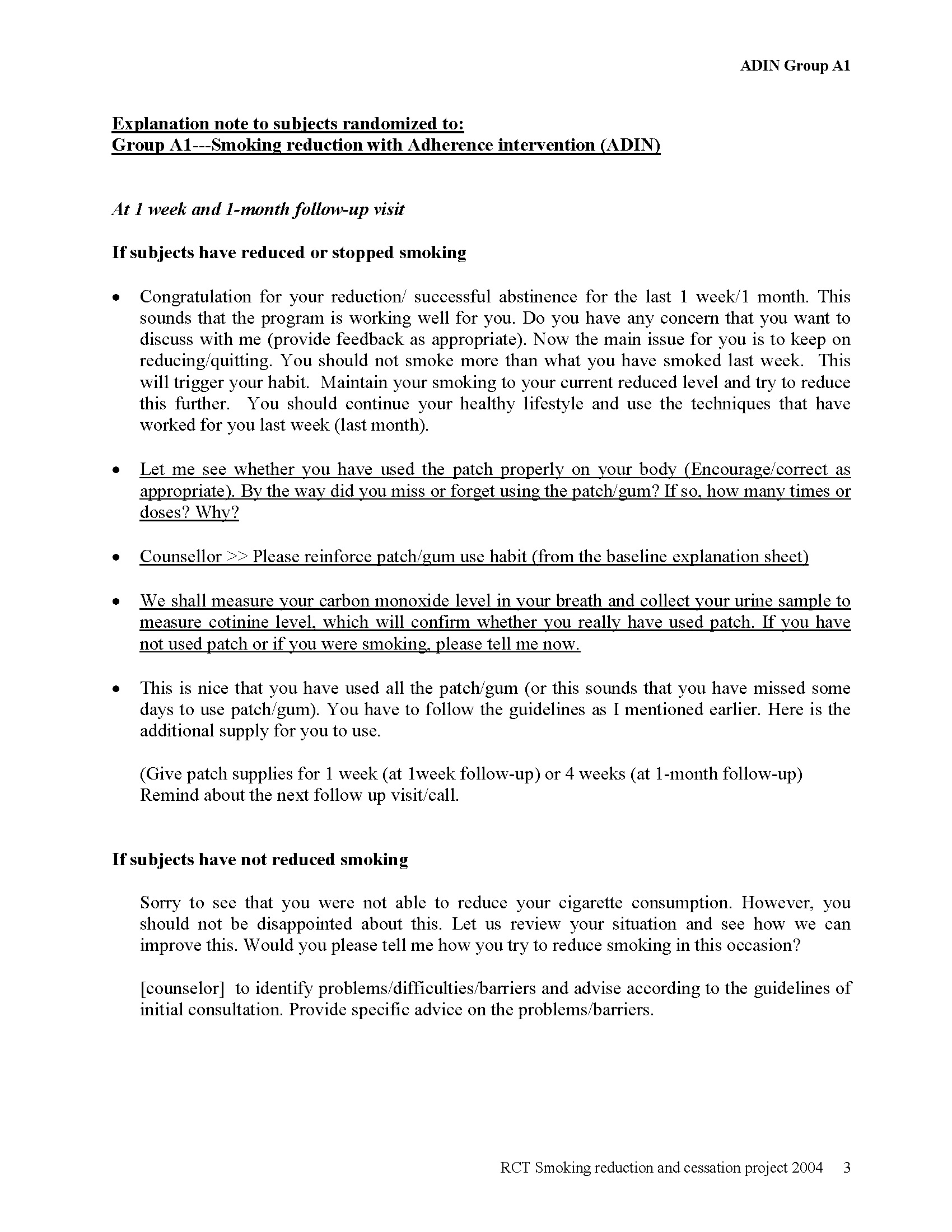

Supplement: Additional file 2: — Appendix B: Manual for behavioral counseling of the intervention Group A1 (Smoking Reduction + Adherence Counselling). [file 12971_2015_34_MOESM2_ESM.docx]
