# Supplementary figures and images for: Effectiveness of smoking reduction intervention for hardcore smokers
Source: Tob Induc Dis. 2015 Apr 2;13(1):9. doi: 10.1186/s12971-015-0034-y (PMC4391680; doi:10.1186/s12971-015-0034-y)

Appendix 3: Manual for behavioral counseling of the intervention Group A2(Smoking Reduction)


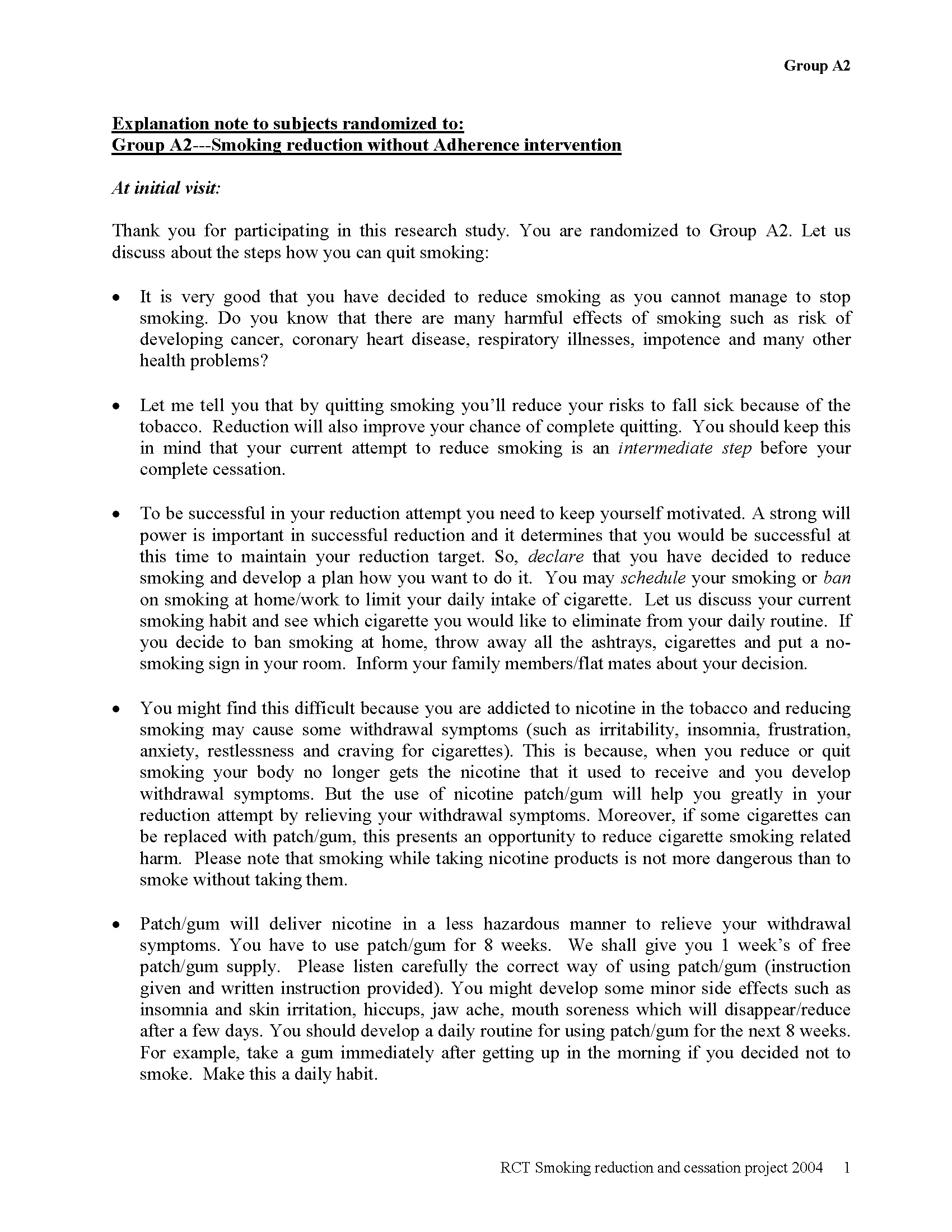


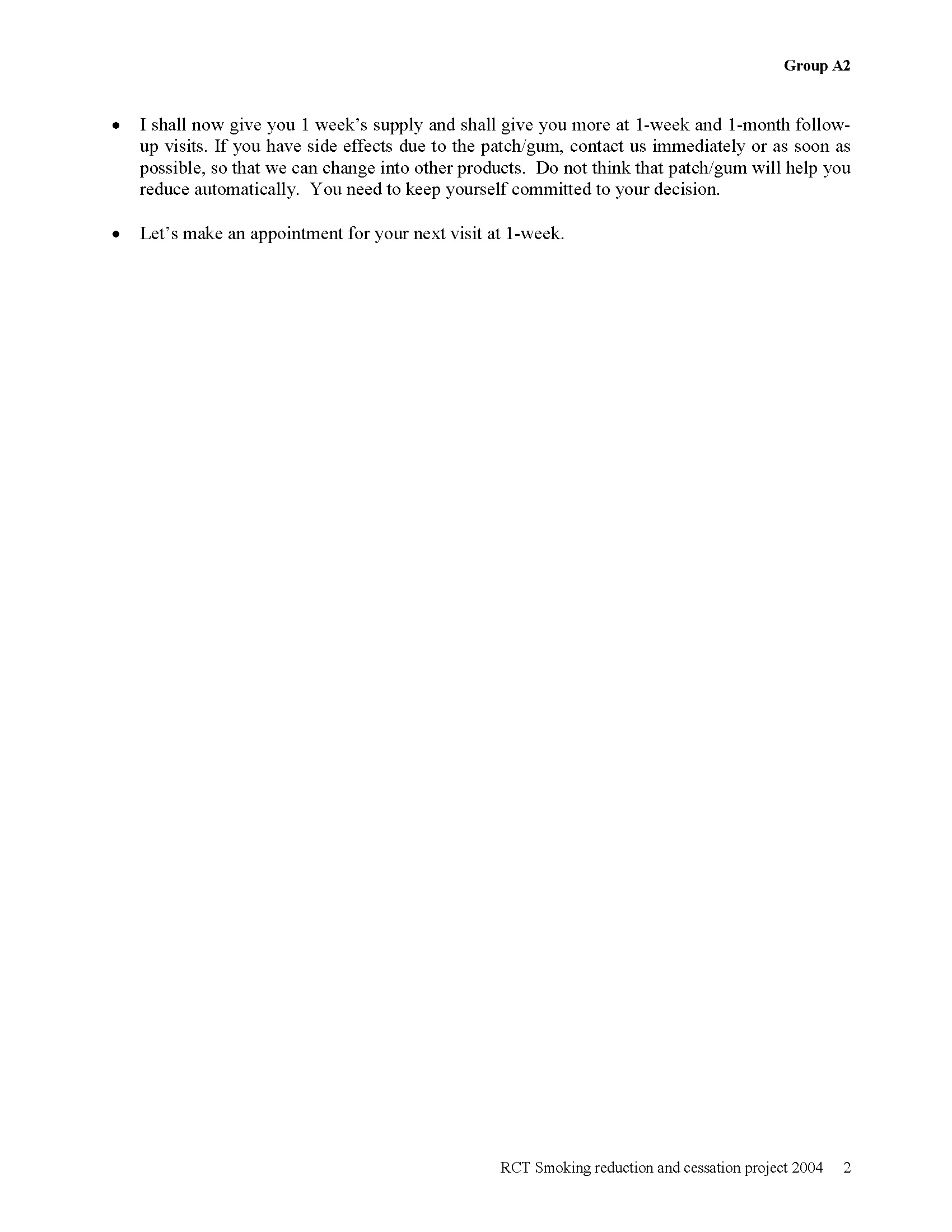


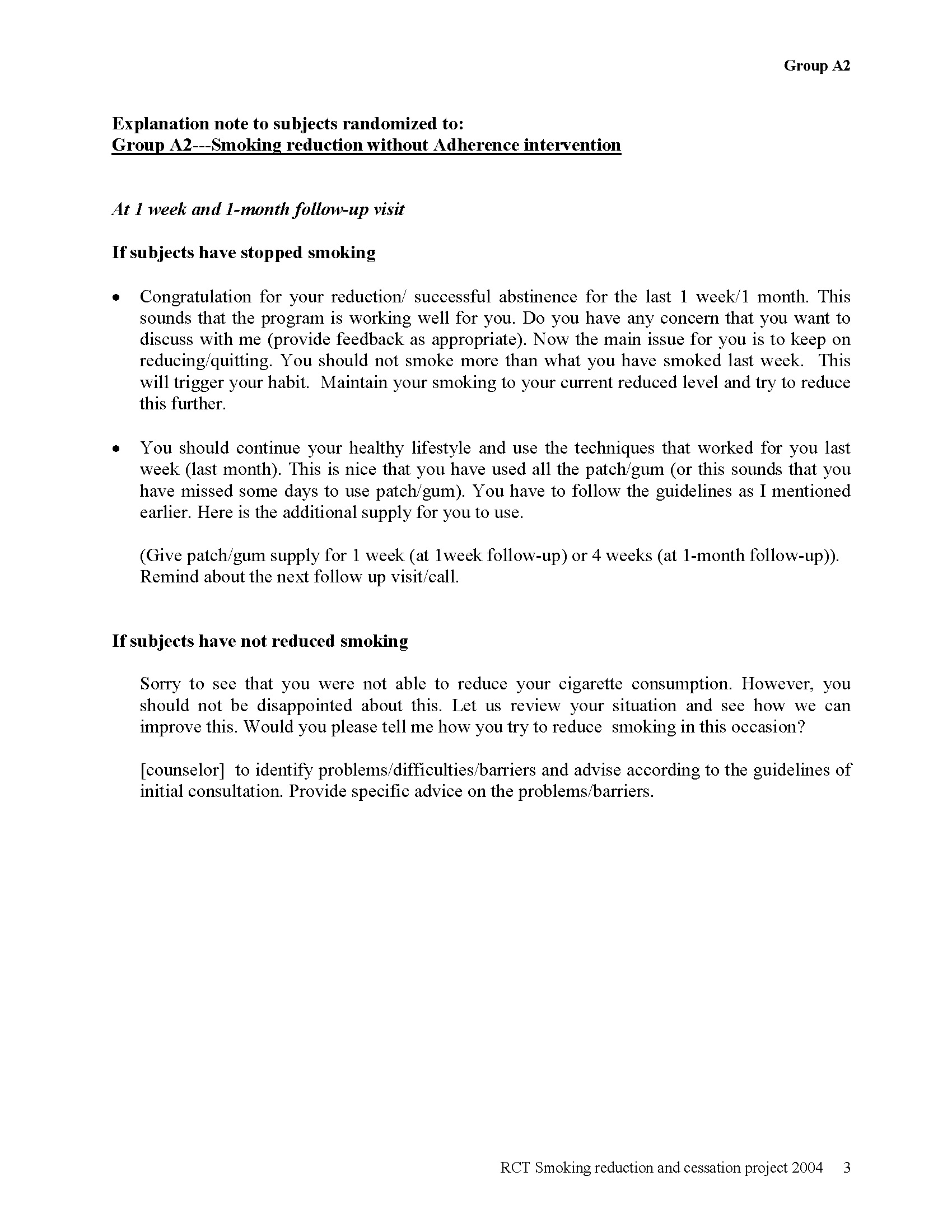

Supplement: Additional file 3: — Appendix C: Manual for behavioral counseling of the intervention Group A2 (Smoking Reduction). [file 12971_2015_34_MOESM3_ESM.docx]
