# Supplementary material for: Effectiveness of smoking reduction intervention for hardcore smokers
Source: Tob Induc Dis. 2015 Apr 2;13(1):9. doi: 10.1186/s12971-015-0034-y (PMC4391680; doi:10.1186/s12971-015-0034-y)
Supplement: Additional file 4: — Appendix D: Baseline characteristics of the participants by hardcore smoking. [file 12971_2015_34_MOESM4_ESM.docx]

Appendix 4: Baseline characteristics of the participants by hardcore smoking

|  | Non-hardcore smokers  (n=969) | Hardcore smokers  (n=184) | p-value |
| --- | --- | --- | --- |
| Age |  |  |  |
| Age > 25 | 927 (95.7) | 184 (100.0) |  |
| Age <= 25 | 42 (4.3) | 0 (0.0) |  |
| Years of regular smoking |  |  |  |
| > 5 years | 948 (97.8) | 184 (100.0) |  |
| <= 5 years | 21 (2.2) | 0 (0.0) |  |
| Number of cigarettes per day |  |  |  |
| >15 | 659 (68.0) | 184 (100.0) |  |
| <=15 | 310 (32.0) | 0 (0.0) |  |
| How many serious attempts (at least 24 hours) have you made at stopping smoking? |  |  |  |
| None | 63 (6.5) | 184 (100) |  |
| 1 attempt | 321 (33.3) | 0 (0) |  |
| 2 to 5 attempts | 479 (49.6) | 0 (0) |  |
| 6 to 10 attempts | 40 (4.1) | 0 (0) |  |
| more than 10 attempts | 62 (6.4) | 0 (0) |  |
| Intervention group, n(%) |  |  |  |
| Intervention (A1+A2) | 777 (80.2) | 150 (81.5) |  |
| Control (B) | 192 (19.8) | 34 (18.5) | 0.68 |
| Age, mean ± SD, years | 42.0±10.7 | 42.4±9.6 | 0.62 |
| Sex, n (%) |  |  |  |
| Male | 798 (82.4) | 147 (79.9) |  |
| Female | 171 (17.6) | 37 (20.1) | 0.43 |
| Marital status, n(%) |  |  |  |
| Married/Cohabiting | 678 (70) | 133 (72.3) | 0.53 |
| Others | 291 (30) | 51 (27.7) |  |
| Education level, n(%) |  |  |  |
| Primary or below | 109 (11.2) | 19 (10.3) |  |
| Secondary | 622 (64.2) | 126 (68.5) | 0.57 |
| Tertiary or above | 238 (24.6) | 39 (21.2) | 0.84 |
| Employment status, n(%) |  |  |  |
| Student (full-time) | 7 (0.7) | 0 (0) | 1.00 |
| Retired | 73 (7.5) | 12 (6.5) | 0.63 |
| Unemployed | 81 (8.4) | 17 (9.2) | 0.62 |
| Currently employed | 807 (83.4) | 155 (84.2) |  |
| Income level, n(%) |  |  |  |
| Over 20k | 469 (49.9) | 84 (47.2) | 0.77 |
| 10-20k | 398 (42.3) | 77 (43.3) | 0.53 |
| Below 10k | 73 (7.8) | 17 (9.6) |  |
| Number of children under 3 at home, n(%) |  |  |  |
| Yes | 83 (8.6) | 20 (10.8) | 0.32 |
| No | 885 (91.4) | 165 (89.2) |  |
| Spouse is a smoker, n(%) |  |  |  |
| Yes | 147 (15.2) | 32 (17.3) | 0.45 |
| No | 820 (84.8) | 153 (82.7) |  |
| Age started smoking, mean ± SD, years | 17.8±4.8 | 17.6±4.7 | 0.67 |
| Years of regular smoking, mean ± SD, years | 24.1±10.8 | 24.7±9.0 | 0.61 |
| Drinking behavior |  |  |  |
| No drinking | 347 (35.9) | 65 (35.3) |  |
| Occasional drinking | 359 (37.2) | 55 (29.9) | 0.82 |
| Frequent drinking | 260 (26.9) | 64 (34.8) | 0.16 |
| Physical exercise in the past month |  |  |  |
| Yes | 453 (46.8) | 75 (40.8) | 0.13 |
| no | 515 (53.2) | 109 (59.2) |  |
